# Dose Rate Effect on Cell Survival in BNCT

**DOI:** 10.3390/cimb45090441

**Published:** 2023-08-23

**Authors:** Katsumi Hirose, Mariko Sato, Koji Ichise, Masahiko Aoki

**Affiliations:** 1 Department of Radiation Oncology, Graduate School of Medicine, Hirosaki University, 5 Zaifu-cho, Hirosaki 036-8562, Japan; s_mariko@hirosaki-u.ac.jp (M.S.); ichise@hirosaki-u.ac.jp (K.I.); maoki@hirosaki-u.ac.jp (M.A.); 2 Southern Tohoku BNCT Research Center, 7-10 Yatsuyamada, Koriyama 963-8052, Japan; 3 Osaka Heavy-Ion Therapy Center, 3-1-10 Otemae, Chuo-ku, Osaka 540-0008, Japan

**Keywords:** boron neutron capture therapy (BNCT), dose rate, borofalan(^10^B)

## Abstract

The output constancy of the accelerator used for boron neutron capture therapy (BNCT) is essential to ensuring anti-tumor efficacy and safety. BNCT as currently practiced requires a wide variety of beam quality assessments to ensure that RBE dose errors are maintained within 5%. However, the necessity of maintaining a constant beam dose rate has not been fully discussed. We therefore clarified the effect of different physical dose rates of the accelerator BNCT on biological effects. SAS and A172 cells exposed to ^10^B-boronophenylalanine were irradiated using a neutron beam (normal operating current, 100 μA) at the Aomori Quantum Science Center. Thermal neutron flux was attenuated to 50.0 ± 0.96% under 50 μA irradiation compared to that under 100 μA irradiation. Cells were given physical doses of 1.67 and 3.36 Gy at 30 and 60 mC, respectively, and survival was significantly increased after 50 μA irradiation for both cell types (*p* = 0.0052 for SAS; *p* = 0.046 for A172, for 60 mC). Differences in accelerator BNCT beam dose rates have non-negligible effects on biological effects. Dose rate fluctuations and differences should not be easily permitted to obtain consistent biological effects.

## 1. Introduction

In Japan, boron neutron capture therapy (BNCT) for recurrent and locally advanced head and neck cancer is covered by the national health insurance system. The boron agent accumulates in tumor cells according to the tumor selectivity of the boron agent, killing cells within the 9 and 4 μm range where alpha rays and lithium recoil nuclei are scattered as a result of the boron–neutron fission reaction. This method is effective in patients with tumor recurrence after radiotherapy and in patients with accumulated toxicity in surrounding normal tissues, such as secondary primary tumors [1]. The X-equivalent dose delivered via BNCT is given mainly by the ^10^B concentration in the tissue, the neutron fluence reaching the tissue, and the relative biological effectiveness (RBE) of the boron agent, which varies from tissue to tissue [2]. Therefore, if a higher accumulation of boron and compound biological effectiveness (CBE) can be ensured in tumor cells, tumor cells could be irradiated with higher doses than normal cells can be, resulting in the sparing of adjacent normal cells. Until now, the most common method has been to use a nuclear reactor as a neutron source. Therefore, it was common for the reactor power level to fluctuate on a daily basis [3]. Reflecting this, to date, there is no broad consensus on how constant the neutron dose rate should be during irradiation in neutron irradiation systems. To irradiate equivalent doses as planned, quality assurance (QA) methods have already been established to maintain beam power constancy within 5% [2]. However, the necessity of maintaining the dose rate is not fully recognized in BNCT, and the existence of accelerator systems equipped with Li targets, which have a characteristic of gradually decreasing output during irradiation [4], has led to a situation in which the necessity of maintaining the dose rate is often disregarded. Even neutron beam output fluctuation during treatment is not considered particularly problematic. As long as the final neutron fluence is given as planned, the planned treatment is judged to have been completed. In glioblastoma clinical trial JG002, 15 patients were treated at the Southern Tohoku BNCT Research Center, and the output level of the neutron generator varied from 76.3% to 95.9% (median, 91.9%), depending on the condition of the device on the day of treatment, due to instability associated with initial equipment assembly errors [5].

On the other hand, Kinashi et al. reported that the D_0_ for the survival curve increases when the neutron beam dose rate is attenuated by one-fifth [6], although these conditions are somewhat different from clinical conditions. Whether or not changes in dose rate under realistic clinical conditions have any significant effect on biological effects is not yet clear.

If this study reveals differences in biological effects depending on the dose rate of the thermal neutron flux, which is clinically possible, the risk of RBE-weighted dose differences would be even larger than currently assumed for different tissue depths. This means that additional considerations would need to be included depending on the depth of the target lesion and organs at risk. In addition, in the operation of irradiation equipment, this could lead to conclusion that fluctuations in neutron flux should not be readily allowed, because allowing easy variations in power output could affect treatment efficacy and safety. This investigation is thus considered quite important for exposing potential clinical problems critical to the current practice of administering BNCT to patients. The objective of this study was therefore to clarify whether or not dose rate has a non-negligible effect on cell survival when output power is halved at thermal neutron beam intensities with neutron fluxes of the level actually used in human clinical practice. Another objective included determining the RBE of the neutron beam used in this study at Aomori Quantum Science Center.

## 2. Materials and Methods

### 2.1. Materials

Fetal bovine serum (FBS) was obtained from Sigma-Aldrich (St. Louis, MO, USA). Phosphate-buffered saline was obtained from Fujifilm (Osaka, Japan). Penicillin and streptomycin were obtained from Gibco-Invitrogen Corp (Carlsbad, CA, USA). Giemsa’s stain solution and 1/15 mol/L phosphate-buffered solution (pH 7.2) were obtained from Nacalai Tesque (Kyoto, Japan). ^10^B-boronophenylalanine (Borofalan(^10^B)) was obtained from Stella Pharma Corporation (Osaka, Japan).

### 2.2. Cell Culture and Growth Conditions

The SAS human tongue squamous cell carcinoma cell line was provided by Riken BioResource Center (Ibaraki, Japan). The A172 human glioblastoma cell line was provided by the Cell Resource Center for Biomedical Research Institute of Development, Aging and Cancer, Tohoku University (Sendai, Japan). SAS cells were cultured in serum-free Dulbecco’s modified Eagle’s medium (DMEM) (Fujifilm) and A172 cells were cultured in serum-free DMEM/Ham’s nutrient mixture F-12 (DMEM/F12 1:1; Fujifilm). All media were supplemented with 10% FBS and 1% penicillin/streptomycin, and cells were maintained at 37 °C in a 5% CO_2_ atmosphere.

### 2.3. X-Irradiation

Cells were exposed to X-rays (6 MV) using a Clinac^®^ iX system linear accelerator (Varian Medical Systems, Palo Alto, CA, USA). Irradiation doses of 1–6 Gy were given at a dose rate of 100 MU/min at room temperature.

### 2.4. Neutron Irradiation

Neutron irradiation was performed using the neutron irradiation system at Aomori Quantum Science Center, which employs an acceleration current of 20 MeV at 100 μA (the standard current) and 50 μA (half the standard power). Irradiation was performed with an accelerator charge of up to 30 mC or 60 mC, depending on the conditions. During cell irradiation, a centrifuge tube containing a cell suspension was placed in a 15 mm tube rotation jig, with irradiation performed while the jig was rotated at 2 revolutions/min.

### 2.5. Measurements for Neutron Flux, Cadmium Ratio, and Gamma

Gold foil and cadmium-covered gold foil for measurement of thermal neutrons and a small rod radiophotoluminescence glass dosimeter (RPLD) element (GD-352M; AGC Techno Glass Corporation, Shizuoka, Japan) for gamma measurement were placed in the rotation jig. Neutron irradiation was applied up to the specified accelerator charge at the specified current. The gold foil was collected, measured with a germanium semiconductor detector, and the number of photons with an energy of 411 keV was counted. RPLD elements were collected after irradiation, removed from the case, preheated at 70 °C for 30 min, and left at room temperature for 30 min. The gamma dose was qualitatively evaluated by reading the fluorescence intensity with a glass dosimeter reader (FGD-1000; AGC Techno Glass Corporation). The gamma dose was qualitatively evaluated by reading the fluorescence intensity with the FGD-1000 glass dosimeter reader.

### 2.6. Cell Clonogenic Assay

SAS or A172 cells were seeded in 100 mm dishes. At 40% confluence, borofalan(^10^B) was added to a final concentration of 40 ppm. After 24 h of incubation, cells were treated with trypsin with 40 ppm of borofalan(^10^B), and then a medium with 40 ppm of borofalan(^10^B) was added. Cells were collected in 15 mm centrifuge tubes, suspended and prepared to the specified cell concentration. Centrifuge tubes were immediately placed in a fixture for neutron irradiation and irradiated with a specified charge of 30 mC or 60 mC by generating neutrons with an accelerator current of 50 μA or 100 μA. After irradiation, cells were collected, seeded in 60 mm dishes, and cultured in an incubator. At 7–8 days after irradiation, cultures were discarded, fixed in methanol and stained with Giemsa stain, and then the number of colonies comprising ≥50 cells was measured using an optical microscope. From this, cell survival was calculated for each condition.

### 2.7. Analysis of RBE_Beam_ and CBE

Considering the equivalent dose D_0.1_ when the surviving fraction is 0.1, the equivalent dose D*_Beam,_* _0.1_ when no borofalan(^10^B) is added is given as follows:

D*_Beam,_* _0.1_ = RBE*_Beam_* · T*_Beam,_* _0.1_ · (d*_th_* + d*_epi_* + d*_fast_* +d*_γ_*) (1)

where RBE*_Beam_* is defined as the biological effectiveness factor of the neutron beam including the dose component, T*_Beam,_* _0.1_ is the irradiation time, and d*_th_* + d*_epi_* + d*_fast_* + d*_γ_* is the physical dose including the dose component due to the neutron beam per unit of irradiation time.

The equivalent dose D*_BNCT,_* _0.1_ when borofalan(^10^B) is added is then given by the following:

D*_BNCT,_* _0.1_ = RBE*_Beam_* · T*_Beam,_* _0.1_ · (d*_th_* + d*_epi_* + d*_fast_* +d*_γ_*) + CBE · d*_Boron_* · T*_Boron,_* _0.1_ (2)

where CBE is defined as the compound biological effectiveness factor of borofalan(^10^B) in terms of boron dose, T*_Boron,_* _0.1_ is the irradiation time, and d*_Boron_* is the physical dose of boron per unit of irradiation time.

From D*_Beam,_* _0.1_ = D*_BNCT,_* _0.1_, CBE is derived as follows:

CBE = RBE*_Beam_* · (T*_Beam,_* _0.1_/T*_Boron,_* _0.1_ − 1) · (d*_th_* + d*_epi_* + d*_fast_* +d*_γ_*)/d*_Boron_* (3)

Note that the cells used in this study remained exposed to borofalan(^10^B) for more than 24 h prior to irradiation, so the cells are assumed to be in equilibrium with the borofalan(^10^B) concentration in the exposed suspension. d*_th_* + d*_epi_* + d*_fast_* + d*_γ_* is 0.108 Gy/min and d*_Boron_* (40 ppm ^10^B) is 0.228 Gy/min, both representing nominal values based on simulation.

### 2.8. Statistical Analysis

All experiments were performed 4 times for physical dose measurements and at least 3 times for the cell clonogenic assay. Results are expressed as mean ± standard deviation. Statistical significance was estimated using Student’s *t*-test. GraphPad Prism version 10.0.0 software (GraphPad Software, Boston, MA, USA) was used for all statistical analyses. Probability values of *p* < 0.05 were considered statistically significant.

## 3. Results

### 3.1. RBE_Neutron beam_ and CBE

**Figure 1:**
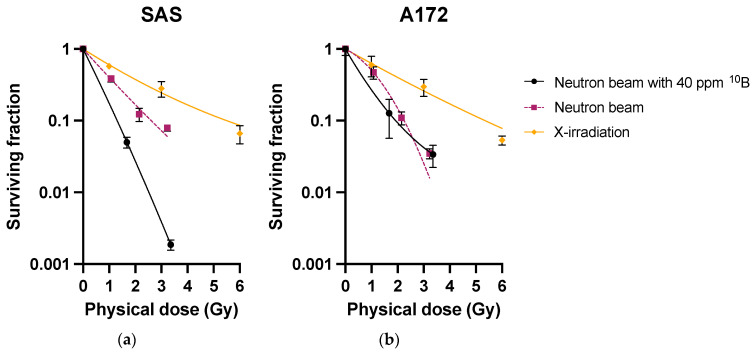
Surviving fraction for deriving *RBE_Beam_ and CBE*. Survival curves for SAS (**a**) and A172 (**b**) are depicted.

Cell viability after X-irradiation and after neutron beam irradiation are shown in Figure 1 and Table 1. For SAS, D_0.1_ from X-irradiation was 5.50 Gy and D_0.1_ from neutron beam irradiation was 2.61 Gy. The RBE*_Beam_* with a mixed beam of neutrons was 2.11. Similarly, for A172 cells, the D_0.1_ from X-irradiation was 5.35 Gy, the D_0.1_ from the neutron beam was 2.25 Gy, and the RBE*_Beam_* was 2.38 (Table 1). Total RBE-weighted dose given to each cell that reached equilibrium in suspension at 40 ppm ^10^B by operating at 100 μA, for 60 mC, was 13.70 Gy-Eq, of which 86.6% was the boron dose for SAS cells. Similarly, for A172 cells, the total RBE-weighted dose was 8.68 Gy-Eq, with 78.9% being from boron (Table 2). Irradiation over 30 min, comparable to that under clinical conditions, would deliver a dose to the tumor greater than the 25 Gy-Eq expected in the clinical setting. Irradiation with this beam, with the dose rate attenuated by half, was thus considered to reproduce dose rate variations similar to those under clinical conditions.

### 3.2. Thermal Neutron Flux and Gamma-Ray Conditions at Half Power

**Figure 2:**
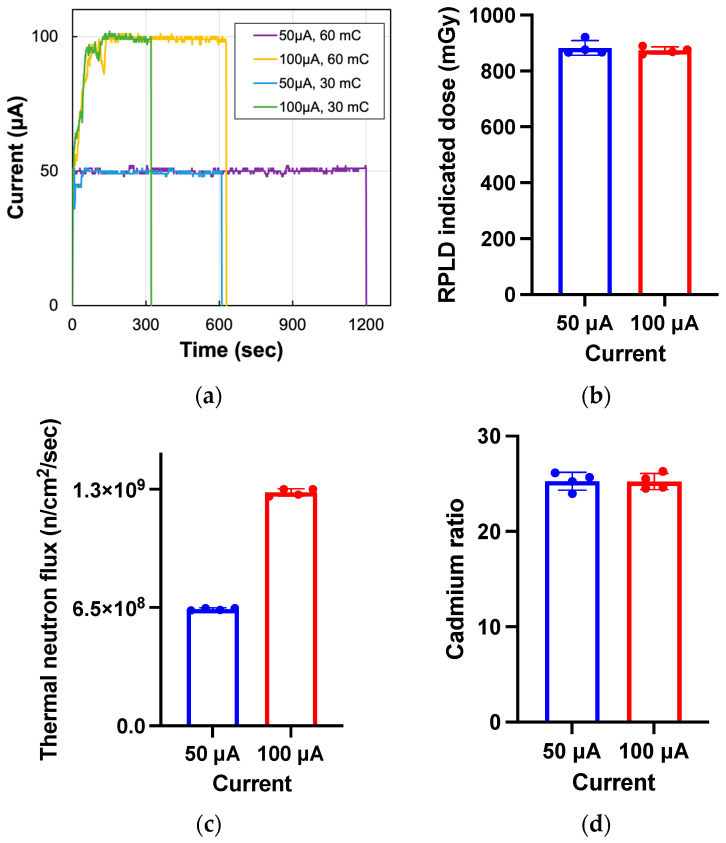
Thermal neutron flux and gamma-ray conditions at half power. (**a**) Example of actual accelerator operating currents. (**b**) Gamma dose measured with the radiophotoluminescence glass dosimeter (RPLD). (**c**,**d**) Thermal neutron flux and cadmium ratio derived from the analysis of gold foil activation with/without a cadmium cover.

Operations at 100 μA, as the standard current, ensured a neutron flux of 1.28 × 10^9^/cm^2^/s, but operation at 50 μA showed that the flux was exactly halved to 6.41 × 10^8^/cm^2^/s. On the other hand, cadmium ratios, reflecting the ratio of thermal to extra-thermal neutrons, were 25.25 ± 0.93 and 25.22 ± 0.83 for 100 μA and 50 μA, respectively, showing no significant difference. Gamma measurements using RPLD also showed no difference, at 882 ± 26 mGy and 873 ± 13 mGy, respectively (Figure 2). These results suggest that thermal neutron flux is reduced by half and the energy spectrum of the neutron and gamma rays included are unchanged under the operation of the accelerator at half-power.

### 3.3. Effect of Beam at Half Power on Cell Survival

**Figure 3:**
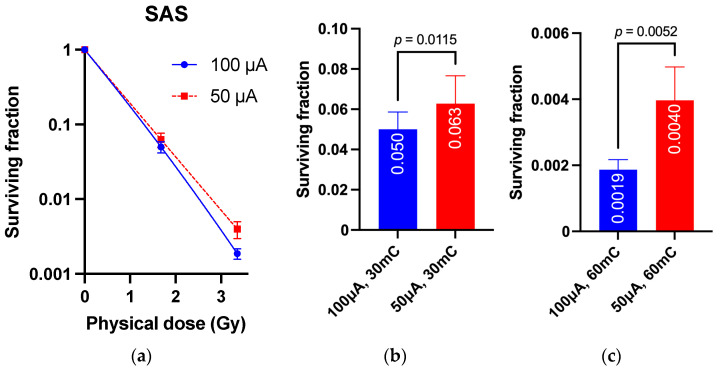
Effect of neutron beam dose rate on surviving fraction for SAS. (**a**) Survival curve. (**b**) Surviving fraction at 30 mC. (**c**) Surviving fraction at 60 mC.

**Figure 4:**
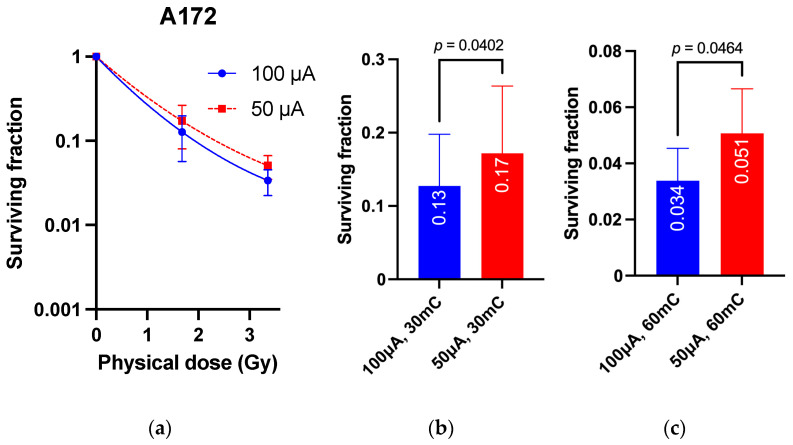
Effect of neutron beam dose rate on surviving fraction for A172. (**a**) Survival curve. (**b**) Surviving fraction at 30 mC. (**c**) Surviving fraction at 60 mC.

SAS or A172 cells were irradiated with accelerator charges at 30 mC and 60 mC using beams at 100 μA and 50 μA, and 1.67 and 3.36 Gy were given as the physical dose to cells at 30 mC and 60 mC, respectively. The survival rates of both cell types were significantly increased after 50 μA irradiation; for SAS cells, the cell survival rate was 1.9 × 10^−3^ ± 2.7 × 10^−4^ vs. 4.0 × 10^−3^ ± 9.1 × 10^−4^ at 60 mC (*p* = 0.0052; Figure 3). For A172 cells, the survival rate was 3.4 × 10^−2^ ± 1.1 × 10^−2^ vs. 5.1 × 10^−2^ ± 1.4 × 10^−4^ at the same 60 mC (*p* = 0.046; Figure 4). These results suggest that differences in the beam dose rate of the BNCT accelerator have non-negligible effects on biological effects.

## 4. Discussion

Dose rate effects have been widely reported for photon radiation therapy [7,8,9,10,11,12]. However, most clinically significant cases have been limited to low-dose exposures. This is because the biological effects of each dose rate only show clear differences at high dose rates, and the dose rate effect of a single fraction is usually negligible for general fractionated irradiations of 2 Gy to several Gy at a time. However, as shown in the figures provided in the report by Matsuya et al., the dose rate effect is not negligible when the dose rate is about 0.18 Gy/min and the dose to the tumor is >10 Gy per fraction [9]. The dose to the tumor is typically ≥20 Gy with >0.3–0.5 Gy/min from one fraction in BNCT. Therefore, dose rate effects that are negligible in conventional irradiation cannot be expected to remain negligible at the dose levels that should be considered clinically relevant for BNCT. Under the doses used in this investigation, the cell survival rates were small for both the 100 μA and 50 μA groups. This implies almost complete cell death under the experimental condition. However, there was a significant difference between the cell survival rates of the 100 μA and 50 μA groups in both cell types. In the clinical irradiation of humans, it is almost impossible to control tumors at similar doses to those used in the experiments, and tumor control may be more greatly influenced by slight differences in biological effects identified in cellular experiments. Additionally, tumor control in humans is not limited to the gross disappearance of tumors, but differences in cell survival are rather meaningful with respect to the risk of recurrence after treatment, which may have a significant impact on patient survival. The high recurrence rate of BNCT for squamous cell carcinoma of the head and neck is a major issue [1,13,14], and it is assumed that a patient’s survival time after treatment is particularly affected by how well surviving cells are retained. Therefore, dose rate fluctuations that affect survival must be appropriately controlled. Considering the current status of BNCT in clinical practice in Japan, the identification of dose rate effects from BNCT in this study will reduce the risks to patients due to over- or under-dosing from the inadvertent handling of dose rates. However, as this study was limited to experiments using an in vitro model, further investigation is required on the impact of dose rates in vivo or based on clinical data.

Currently, accelerator-based BNCT devices are being commercialized. Beginning with NeuCure^®^ manufactured by Sumitomo Heavy Industries as the first system to achieve regulatory approval in Japan [15], accelerator-based neutron irradiation devices manufactured by CICS (Cancer Intelligence Care Systems, Inc., Tokyo, Japan) are currently undergoing clinical trials in Japan with the aim of obtaining regulatory approval [16,17]. Other neutron irradiation systems manufactured by Neutron Therapeutics [18] and developed by the University of Tsukuba and Nagoya University are also under development for regulatory approval [19,20]. The ratio of dose components that make up the beam has been shown to not be very different between these devices, and the profile of neutron fluence in the depth direction in a water standard phantom is not significantly different if adjusted for the time of irradiation. Beam similarity between instruments has been discussed only in terms of beam profiles, and the discussion of dose rates has been very reluctantly carried out. However, some differences in neutron flux are seen between instruments, to a greater or lesser extent. For example, the neutron flux generated by the Neutron Therapeutics accelerator system is double that shown by Sumitomo NeuCure^®^ (Sumitomo Heavy Industries, Ltd., Tokyo, Japan) [15,18]. Ignoring the dose rate argument is ultimately detrimental to patients in clinical practice and should never occur for ethical reasons. The present results show that different dose rates produce significant differences in biological effects in real-world clinical neutron fluxes. Accordingly, the difference in dose rates between the two previously mentioned instruments is not likely to be negligible. In the future, setting up a step to examine and optimize the dose prescription to be used for each device will be inevitably required for each disease, taking into account the fact that the biological effects on tumors and normal tissues differ from device to device.

RPLDs are made of phosphate glass, which is less sensitive to thermal neutrons than ordinary TLDs are, and has been shown to be as sensitive to gamma radiation as are custom-made TLDs enclosed in a quartz glass case in a typical neutron field [21]. On the other hand, concerns have been raised about the effect on readings due to the capture reactions of thermal neutrons with the elements O, P, Na, Al, and Ag, all of which are constituent elements of RPLDs. Although the evaluation of the effect of gamma rays is possible solely through an exclusion of the effects of neutrons via covering them with a ^6^LiF neutron-shielding material, the necessary space for the thickness of ^6^LiF in the present irradiation system is difficult to secure. However, considering the results of thermal neutron flux and cadmium ratio due to the use of gold foil and cadmium, the beam was generally unchanged as a spectrum, and the effects of variations in gamma ray readings of the RPLD were not overly large. The present experiment using a beam with the sufficient flux being halved could therefore be undertaken without significant spectral fluctuations.

## 5. Conclusions

The present results indicate that differences in accelerator BNCT beam dose rates have non-negligible biological effects. At present, dose rate fluctuations and differences should not be readily permitted in order to obtain the same biological effects. From a clinical perspective, a reduction in thermal neutron flux may result in even larger biological differences than those with the apparent dose given via dose calculation. This implies that in the treatment of patients, avoiding deviations from the planned setup and deviations from the plan during irradiation is extremely important to ensure that doses to the tumor and normal tissues are delivered as planned. If dose rate fluctuations during treatment are to be permitted in the future, a detailed study is needed to determine safety margins for ranges of fluctuations and the relationship between dose rate and charge that will produce the same biological effect.

## Figures and Tables

**Table 1:** RBE*_Beam_* and CBE of cells when irradiated with 100 μA for 60 mC.

Cell Type	D_0.1_ for X-Irradiation (Gy)	D_0.1_ for Neutron Beam (Gy)	RBE*_Beam_*	CBE
SAS	5.50	2.61	2.11	5.23
A172	5.35	2.25	2.38	2.97

**Table 2:** Dose composition of the RBE-weighted dose given to cells with 40 ppm ^10^B irradiated with 100 μA for 60 mC.

Cell Type	Thermal Neutron (Gy-Eq)	Epithermal Neutron (Gy-Eq)	Fast Neutron (Gy-Eq)	Gamma (Gy)	^10^B Dose (Gy-Eq)	Total RBE Dose (Gy-Eq)
SAS	0.41	0.01	0.72	0.70	11.87	13.70
A172					6.85	8.68

## Data Availability

Data sharing is not applicable to this article.
